# Stroke and Risk Factors in Antiphospholipid Syndrome

**DOI:** 10.3390/jpm14010024

**Published:** 2023-12-24

**Authors:** Yangyi Fan, Yicheng Xu, Sifan Zhang, Xiaodong Song, Zunjing Liu, Wenjun Tu, Chun Li

**Affiliations:** 1 Department of Neurology, Peking University People’s Hospital, Beijing 100044, China; fanyangyi@bjmu.edu.cn (Y.F.); songxiaodong@pkuph.edu.cn (X.S.); liuzunjing@bjmu.edu.cn (Z.L.); 2 Department of Neurology, Aerospace Center Hospital, Beijing 100049, China; 3 Peking University People’s Hospital, Beijing 100191, China; 1910301206@pku.edu.cn; 4 Department of Neurology, The First Affiliated Hospital of Chongqing Medical University, Chongqing 400042, China; 5 Department of Neurosurgery, Tiantan Hospital, Beijing 100050, China; 6 Department of Rheumatology and Immunology, Peking University People’s Hospital, Beijing 100044, China

**Keywords:** stroke, antiphospholipid, risk factor, stroke recurrence

## Abstract

Stroke is considered one of the most common and life-threatening manifestations of antiphospholipid syndrome (APS), which leads to high mortality and permanent disability. This study investigated the prevalence and the potential risk factors of stroke in APS. We enrolled 361 APS patients retrospectively from 2009 to 2022 at Peking University People’s Hospital. Stroke was found in 25.8% (93/361) of the participants. The multivariate logistic regression showed that hypertension, diabetes, livedo reticularis, and other central nervous system involvements were significant related factors for stroke. The use of hydroxychloroquine appeared to relate to a lower incidence of stroke. During a median follow-up of 3.0 years, 11.8% (11/93) of the individuals with a previous stroke developed stroke recurrence, and thrombocytopenia seemed to be a predictor of stroke recurrence.

## 1. Introduction

Antiphospholipid syndrome (APS) is an autoimmune disorder featuring thrombosis (arterial, venous, or both), obstetric morbidity, and the presence of persistent antiphospholipid antibodies (aPLs) in medium to high titers [1,2,3]. The estimated incidence and prevalence range between five cases per 100,000 per year and 40 to 50 per 100,000 [4]. Due to the thrombophilia caused by the antiphospholipid antibodies, recurrent thrombotic events in both arteries and the venous system are a main clinical feature of APS. The cerebral vascular system is frequently involved, which accounts for nearly half of the arterial events in APS, and cerebrovascular events are usually the life-threatening complications of APS. Ischemic stroke or transient ischemic attack (TIA) were found to have initial presentation in almost 30% of APS adults [5]. In a European cohort of 1000 patients with APS, cerebral infarction was reported in 19.8% of the study population and accounted for 11.8% of all deaths during a 10-year follow-up period [6]. In addition, 17% of strokes and 12% of TIA in patients younger than 50 years old were reported to be associated with aPLs [7], suggesting that APS is a significant contributor to stroke in younger patients. In venous circulation, although cerebral vein thrombosis (CVT) is a less common presentation, it has been estimated that about 80% of CVT cases associated with APS represent the initial clinical manifestation of APS [8].

Concerning the mechanisms of stroke in APS, thrombosis is considered the most common pathological process. Intracranial artery occlusions and stenosis are present in 50% of patients with APS and stroke, with the middle cerebral artery mostly involved, in which patients usually present hemiparesis, dysarthria, hemidysesthesia, and aphasia if the lesion is in the dominant hemisphere. Cardioembolic mechanisms are another reason due to left-sided cardiac valve abnormalities, such as irregular valve thickening due to immune complex deposition, vegetations, and valve dysfunction. Chronic occlusive vasculopathy affecting small-to-medium-sized intracerebral arteries is also observed, leading to lacunar or subcortical strokes [9]. Carotid or vertebral artery dissection, Sneddon’s syndrome, and Moyamoya disease are also described in stroke with APS.

Considering the high mortality and severe permanent disability caused by stroke, especially in a large population base in China [10,11], identifying patients with APS at high risk of stroke and stroke recurrence is urgently needed and remains a challenge for physicians. This study aimed to analyze the prevalence, clinical features, and risk factors of stroke in a cohort of APS patients from China, and to evaluate the rate and predictive factors of stroke recurrence during follow-up, which may provide evidence for prevention and therapy strategies for stroke in APS.

## 2. Materials and Methods

### 2.1. Patients and Baseline Data Collection

We retrospectively enrolled patients diagnosed with definitive APS who visited Peking University People’s Hospital between 1 January 2009 and 1 July 2022 (both inpatients and outpatients in the clinic). The diagnosis of APS was made by the attending physicians according to the Sydney-revised Sapporo criteria [1]. In the Sydney-revised Sapporo criteria, APS is present if at least one of the clinical criteria and one of the laboratory criteria are met. The clinical criteria include vascular thrombosis and pregnancy morbidity, while the laboratory criteria include three antiphospholipid antibodies: lupus anticoagulant (LA), anticardiolipin (aCL) antibody, and anti-β2 glycoprotein I antibody (aβ2GPI). The presence of either of the antibodies should be detected on two or more occasions with an interval of at least 12 weeks. The following characteristics were cause for exclusion: (1) stroke happened before the diagnosis of APS; (2) stroke coexisted with other coagulation disorders (malignancy, hematological disorders, or severe liver disease); (3) incomplete clinical medical record data. This study was approved by the Peking University People’s Hospital Ethics Committee (2019PHB252) and informed consent was waived according to relevant regulations. 

The following demographic clinical information was obtained: age, gender, disease duration, the coexistence of other autoimmune diseases, thrombotic events, and obstetric complications, as well as APS non-criteria manifestations including livedo reticularis, APS nephropathy, diffuse alveolar hemorrhage, and thrombocytopenia (platelet counts < 100 × 10^9^/L) at diagnosis. Other neurological manifestations of APS were evaluated by experienced neurologists according to relevant criteria (cognitive impairment, migraine, epilepsy and seizure, multiple-sclerosis-like syndrome, transverse myelitis, neuro-ophthalmologic involvement, movement disorders, and psychiatric disorders). 

Traditional cardiovascular risk factors recorded included hypertension, diabetes mellitus, hyperlipidemia, smoking, obesity (body mass index (BMI) > 30 kg/m^2^), hyperuricemia, chronic kidney disease, and atrial fibrillation. Treatments with anticoagulants (warfarin, new direct oral anticoagulants, or low-molecular-weight heparin), antiplatelets (aspirin, clopidogrel, cilostazol, and tegrilol), corticosteroids, hydroxychloroquine (HCQ), and immunosuppressive agents were also recorded.

### 2.2. Assessment of Stroke

Stroke was identified by two experienced neurologists based on patient history, clinical manifestations, and an objective verification of neuroimaging findings (magnetic resonance imaging (MRI) or computed tomography (CT)). Stroke subtypes were classified as ischemic stroke, TIA, CVT, intracranial hemorrhage (ICH), or subarachnoid hemorrhage (SAH) according to the 2013 American Heart Association/American Stroke Association updated definition of stroke, and confirmed by either CT or MRI with magnetic resonance angiography/venography. Any inconsistency in the definition of stroke was resolved by a consultation with a senior neurologist. 

### 2.3. Detection of aPLs and aGAPSS

The aCL and aβ2GPI were detected using ELISA kits (EUROIMMUN, Luebeck, Germany). Samples with aCL > 12 IU/mL or aβ2GPI > 27 RU/mL were considered positive. The LA assay was performed according to the recommended criteria from the ISTH subcommittee on lupus anticoagulant/phospholipid-dependent antibodies, i.e., the dRVVT ratios (LA1 screen/LA2 confirmation), with >1.2 being considered positive for LA activity. The aGAPSS was calculated by adding corresponding points to the risk factors: 3 for hyperlipidemia, 1 for arterial hypertension, 5 for aCL, 4 for aβ2GPI, and 4 for LAC [12].

### 2.4. Follow-Up

Follow-up was started after the APS patients were enrolled and was performed every three months via clinic visits or telephone interviews. It ended at either the time of a stroke recurrence, death from any cause, or the last follow-up visit (1 July 2023). Recurrent stroke was identified by a neurologist according to the clinical criteria based on typical clinical syndrome and radiological evidence (MRI or CT).

### 2.5. Statistical Analysis

All statistical analyses were performed using IBM SPSS Statistics version 22.0 (IBM Corp, Armonk, NY, USA). A 2-sided *p*-value less than 0.05 was considered to indicate statistical significance. Categorical variables were presented as counts (percentages). Continuous variables were expressed as mean (standard deviation) if normally distributed, or median (interquartile range) otherwise. Patients with and without stroke were compared regarding demographics and laboratory parameters. For categorical variables, Fisher’s exact test and chi-square test were employed to analyze group differences. For continuous variables, Student’s t-test (if normally distributed) and Mann–Whitney U test (if abnormally distributed) were applied to identify differences between groups. Univariate logistic regression was used to explore the potential related factors, and variables exhibiting significant differences (*p* ˂ 0.05) were included in the binary logistic regression models. Multiple logistic regression models were applied to determine the 95% confidence intervals (95% CIs) and odds ratios (ORs).

Univariate logistic regression analysis was applied to explore the independent predictors of recurrent stroke. We set the interval between the first recurrence of stroke and the stroke initially diagnosed in the APS patient as the time to recurrence.

## 3. Results

### 3.1. Clinical Characteristics of the APS Group

- A total of 361 patients were enrolled in the final analysis, with 279 females and 82 males, aged 44.7 (16.8) years (range 16–88 years). While 51.2% (185/361) of the patients had primary APS, secondary APS accounted for 48.8% (176/361) of the cohort. Systemic lupus erythematosus was the most commonly combined autoimmune disease (75.0%, 132/176). A total of 63.2% (228/361) of the patients presented with isolated thrombotic APS, 28.0% (101/361) of them presented with isolated obstetric APS, and 8.9% (32/361) with both.
- Stroke was found in 25.8% (93/361) of the whole cohort. Among the patients, ischemic stroke was the most frequent (23.5%, 85/361), followed by TIA (1.7%, 6/361) and cerebral venous sinus thrombosis (1.1%, 4/361). Among ischemic stroke, the percentage of acute ischemic stroke was 35.3% (30/85). There were two patients with both ischemic stroke and TIA and one patient with both ischemic stroke and venous sinus thrombosis, while ICH occurred in one patient.
- In the samples we collected, 34.1% (123/361) had central system involvement. Except for stroke, the neuropsychiatric abnormalities were the second-most common manifestations (19.5%, 24/123), followed by seizures and epilepsy (14.6%, 18/123), migraine (11.3%, 14/123), cognitive impairment (5.7%, 7/123), optic neuritis (4.9%, 6/123), multiple-sclerosis-like disease (2.4%, 3/123), and movement disorders (2.4%, 3/123).

### 3.2. Distribution of Risk Factors and Treatment between Patients with and without Stroke

Compared with patients without stroke, patients with stroke were older (51.0 (16.8) vs. 42.5 (15.8) years, *p* ˂ 0.001), with a higher percentage of males (31.2% vs. 19.8%, *p* = 0.024) and secondary APS (60.0% vs. 44.8%, *p* = 0.010). They more often presented as simple thrombotic APS (83.9% vs. 56.0%, *p* ˂ 0.001) but the frequency of the extracranial thrombotic events did not differ significantly between the stroke group and the non-stroke group. The disease duration was comparable between the two groups. In terms of clinical manifestations, stroke patients were more likely to have other central nervous system involvement (32.3% vs. 11.2%, *p* ˂ 0.001), livedo reticularis (8.6% vs. 1.9%, *p* = 0.003), hypertension (50.5% vs. 23.1%, *p* ˂ 0.001), diabetes (24.7% vs. 3.0%, *p* ˂ 0.001), and hyperlipidemia (24.7% vs. 11.9%, *p* = 0.003). Consequently, the participants with stroke had higher aGAPSS scales than those without stroke (10.0 (7.0, 13.0) vs. 9.0 (5.0, 13.0), *p* = 0.022). When it came to the laboratory tests, the platelet levels and the percentage of thrombocytopenia were significantly higher in the stroke group than in the non-stroke group. Coagulation parameters, including the prothrombin (PT), activated partial thromboplastin (APTT), D-dimer, and the percentage of low Complement 3 and Complement 4, did not differ significantly between the two groups. The frequency of the three criteria antibodies did not differ significantly between the patients with stroke and those without stroke. Furthermore, there was no significant difference in the percentage of triple aPLs positivity between the two groups (Table 1).

Univariate logistic regression revealed that male sex (OR: 1.838, 95%CI: 1.080–3.129, *p* = 0.025), increasing age (OR: 1.031, 95%CI: 1.017–1.047, *p* ˂ 0.001), other comorbid immune disorders (OR: 1.867, 95%CI: 1.155–3.017, *p* = 0.001), and other central nervous system involvements (OR: 3.778, 95%CI: 2.121–6.729, *p* ˂ 0.001) increased the risk of stroke. The aGAPSS score was a significant associated factor for stroke (OR: 1.068, 95%CI: 1.006–1.135, *p* = 0.032), and hypertension (OR: 3.395, 95%CI: 2.068–5.574, *p* ˂ 0.001), diabetes (OR: 10.679, 95%CI: 4.579–24.901, *p* ˂ 0.001), hyperlipidemia (OR: 2.423, 95%CI: 1.332–4.409, *p* = 0.004), livedo reticularis (OR: 4.951, 95%CI: 1.577–15.583, *p* = 0.006), and thrombocytopenia (OR: 1.725, 95%CI: 1.071–2.779, *p* = 0.025) were also significantly related with the higher risk of stroke. But the BMI index, smoking, chronic kidney disease, hyperuricemia were not significantly related factors of stroke, and neither the single aPL positivity nor the triple aPL positivity was associated with stroke risk. In terms of treatment, the use of HCQ (OR: 0.441, 95%CI: 0.271–0.717, *p* = 0.001) and statins (OR: 2.393, 95%CI: 1.153–4.968, *p* = 0.019) was significantly associated with stroke, while antiplatelet and anticoagulant therapy did not significantly affect the incidence of stroke (Table 2).

In the multivariate logistic regression model (model 1), only hypertension (OR: 2.201, 95%CI: 1.184–4.089, *p* = 0.013), diabetes (OR: 6.185, 95%CI: 2.441–15.676, *p* ˂ 0.001), and livedo reticularis (OR: 5.027, 95%CI: 1.341–18.851, *p* = 0.017) were found to be significantly associated with increased stroke risk. None of the treatments with anticoagulation, antiplatelet drugs, HCQ, statins, or immunosuppressants appeared to significantly reduce the incidence of stroke (Table 3). In another model, which included the aGAPSS score but excluded hypertension and hyperlipidemia (model 2), diabetes (OR: 6.004, 95%CI: 2.385–15.111, *p* ˂ 0.001) and livedo reticularis (OR: 5.338, 95%CI: 1.491–19.106, *p* = 0.010) were revealed as significant associated factors for stroke, and hydroxychloroquine appeared to be related to a decreased risk of stroke (OR: 0.549, 95%CI: 0.316–0.952, *p* = 0.033) (Table 3).

### 3.3. Follow-Up and Stroke Recurrence

Over a median follow-up period of 3.0 (1.0,6.0) years, ranging from 0.5 to 15.0 years, 11.8% (11/93) of the APS patients with stroke experienced recurring strokes despite undergoing treatment. Among these instances, 10 cases manifested as acute ischemic strokes, while the remaining case was attributed to intracranial hemorrhage. The case of the intracranial hemorrhage was a 42-year-old woman who had both thrombotic and obstetric manifestations. She received both aspirin and warfarin treatment and had intracranial hemorrhage after 7.6 years of enrollment. The international standardized ratio (INR) of PT was 3.5 when the hemorrhage occurred. During the follow-up period, there were five new-onset stroke events in the patients without stroke at enrollment: four cases were acute ischemic stroke, while one case was intracranial hemorrhage. The univariate regression demonstrated that thrombocytopenia (HR: 0.082, 95%CI: 0.010–0.673, *p* = 0.020) was a significant independent predictor of stroke recurrence (Table 4).

## 4. Discussion

In this study, we found that stroke occurred in 25.8% of the whole study population, and ischemic stroke was the most common type of stroke. This is higher than the results reported in a European cohort of 1000 patients [6]. The multivariate analysis revealed that hypertension, diabetes, and other CNS involvements were independent risk factors of stroke in APS. Furthermore, patients with stroke in APS showed a high rate of recurrence (11.8%) despite ongoing therapy, and thrombocytopenia may be a predictor of stroke recurrence.

A positive correlation between the presence of cardiovascular risk factors and an elevated thrombotic risk was observed in patients with APS and aPLs carriers. In a prospective study involving 404 APS patients and aPLs carriers, hypertension and hypercholesterolemia were identified as risk factors for arterial thrombosis [13]. Another retrospective study encompassing 99 aPLs carriers revealed that hypertension, smoking, hyperlipidemia, and diabetes were the most significant predictors of thrombosis [14]. Similarly, smoking and hypertension were identified as independent risk factors for thrombosis in aPLs carriers in a retrospective study which enrolled 138 subjects [15]. Consistent with the preceding findings, our study confirmed that hypertension and diabetes were notably associated risk factors for stroke in individuals with APS. This underscores the substantial role of traditional cardiovascular risk factors in the occurrence of stroke within the APS population and aPLs carriers.

Livedo reticularis stands out as a non-criteria manifestation of APS and represents the most prevalent cutaneous symptom, stemming from the constriction of medium and small arteries at the dermis–subcutis interface. Herein, we found livedo reticularis to be associated significantly with an increased risk of stroke, aligning with the previous studies [16]. The present findings underscore the potential of livedo reticularis as an indicative marker for an escalated stroke risk in APS, emphasizing the imperative need for intensified monitoring and follow-up. Previous studies have illustrated non-inflammatory thrombotic vasculopathy involving small- and medium-sized cerebral arteries, akin to the pathological observations in dermal arteries. The conjecture that parallel thrombotic processes manifest in both cutaneous and central nervous system domains was supported by the connection of stroke to the presence of livedo reticularis. But the specific mechanism is still unknown.

In our study, central nervous manifestations other than stroke were found in 34.1% of the population. It was not unexpected to find a correlation between other CNS involvements and stroke in APS in the univariate regression, as there has been evidence found regarding the underlying interconnected pathological mechanisms. For instance, microthrombosis-induced ischemia or thromboembolic events lead to vascular epilepsy in APS [17], and small vessel ischemic damage associated with aPLs may be responsible for the cognitive impairment in APS, which has been verified by structural magnetic resonance imaging [18]. In addition, migraine, particularly with aura, has been identified as a risk factor for stroke, and data from certain cohorts have also indicated migraine as a possible risk factor for stroke in aPLs-positive patients. The aPLs-induced cerebral vascular sludging or microthrombosis might be underlying common pathological mechanisms for migraine and stroke [19]. But due to the limited cases, it remains challenging to identify the definite relation between stroke, other CNS manifestations, and the potential common mechanisms in APS.

It is widely acknowledged that the thrombotic risk increases with the number of positive aPLs, particularly in individuals exhibiting positivity for all three criteria aPLs (triple aPL positivity) [15]. A previous study reported aβ2GPI positivity to be associated with a higher risk of stroke [20]. However, in our study, neither the presence of any single aPL positivity nor triple aPL positivity demonstrated an association with the occurrence or recurrence of stroke. Our results are consistent with the findings in a retrospective study containing 120 cases of APS, in which aPL profiles were not associated with acute cerebrovascular diseases [21]. In another study focusing on the profiles of aPLs and the clinical phenotypes of APS, none of the three criteria antibodies was associated with CNS manifestations [22]. There might be several reasons for that. Firstly, only the three criteria antibodies were included into the analysis, while a profile of noncriteria antibodies participate in the pathogenesis of thrombosis in APS [22]. Secondly, although it is generally considered that aPLs contribute to stroke pathogenesis through several mechanisms, in the “two hit” hypothesis of thrombosis in APS, the presence of aPLs alone is not sufficient to cause pathology. The prodromal underlying endothelial damage and/or disturbance of the redox balance in the vascular system, which could be caused by multiple cardiovascular risk factors, constitute a “first hit”, and the thrombophilia induced by aPLs acts as a “second hit” to assert the pathogenicity for thrombosis [23]. Hence, it is reasonable that the aPLs were not significant factors associated with stroke in our results.

Based on these findings, we can draw a conclusion that multiple factors participate in the pathogenesis of stroke in APS patients in different links, including the traditional cardiovascular risk factors, other central nervous involvement, and a spectrum of antiphospholipid antibodies, including both criteria and non-criteria antibodies. The existing aGAPSS score, which is used to assess the risk of thrombosis in APS, does not seem to cover all of the factors. Well-designed prospective studies with more comprehensive information included are needed to set up a more precise predictive model for stroke risk in APS.

In our cohort, recurrence occurred in 11.8% of the individuals with stroke. The recurrence rate was higher than that in the Euro-phospholipid cohort [5], but comparable with those found in other long-term studies [21,24]. The univariate analysis showed thrombocytopenia to be associated with a significantly decreased risk of stroke recurrence, contrary to the former study [15]. We attributed this inconsistency to the bias caused by the limited samples. Another reason was the fact that the detailed mechanisms of the aPLs contributing to thrombocytopenia and thrombosis are still not fully understood.

Regarding the treatments for stroke in APS, anticoagulation with warfarin or other vitamin K antagonists (VKA) is generally considered as the cornerstone of APS-associated IS/TIA [25]. But the optimal INR range for APS patients with stroke is still undefined. The choice of dual antiplatelet therapy or combined anticoagulant–antiplatelet therapy for APS-associated arterial thrombosis is of interest and a meta-analysis revealed higher effectiveness of dual antiplatelet therapy compared to single antiplatelet therapy and combined VKA–antiplatelet therapy compared to VKA alone [26], but high-quality studies with large samples are lacking to verify the efficiency. In the present study, we did not find a significant protective role of antiplatelet drugs, anticoagulation, or combined anticoagulant–antiplatelet therapy in stroke or stroke recurrence. Detailed information about the treatments, such as the duration of anti-platelet drugs or anticoagulation, and the INR value, were lacking. Hence, it is hard to obtain enough evidence to find the optimal treatment. The optimal antithrombotic strategy still requires exploration in further studies with detailed treatment monitoring. Otherwise, we found that HCQ might be a protective factor for stroke in our cohort. The protection of HCQ against thrombosis in APS patients has been reported in two studies of non-randomized cohorts with thrombotic primary APS [27]. It is believed the protective function of HCQ is connected with a reduction in aPLs titers [28], as well as its pleiotropic, antithrombotic, and immunoregulatory effects. The role of hydroxychloroquine in preventing stroke in APS needs to be confirmed through further, appropriately designed studies, and it might offer another strategy for stroke treatment in APS.

There are several limitations to our research. Firstly, the heterogeneity of the study population in this retrospective research led to an inevitable bias of the data as well as incomplete information for further analysis, such as the difference in the follow-up duration and lacking information regarding the duration between APS being diagnosed and the incidence of stroke. Furthermore, only the criteria antibodies were included; some non-criteria antibodies, which have been reported to be associated with the risk of thrombosis in APS, such as anti-PS/PT antibodies [29], were not tested. Thirdly, the monitoring for antiplatelet and anticoagulation activity was lacking in some patients, and this led to difficulty assessing the effectiveness of the treatments.

## 5. Conclusions

The potential risk factors for stroke in APS include hypertension, diabetes, livedo reticularis, and other CNS manifestations. Thrombocytopenia relates to the risk of stroke recurrence. The risk of stroke in APS could be minimized by controlling modifiable cardiovascular risk factors. HCQ may be effective in protecting against stroke in individuals with APS.

## Figures and Tables

**Table 1:** Demographic, clinical, and laboratory characteristics of the study population.

Variables	All Cases (n = 361)	With Stroke (n = 93)	Without Stoke (n = 268)	*p*
Male gender, n (%)	82 (22.7)	29 (31.2)	53 (19.8)	**0.024**
Age (years), mean (SD)	44.7 (16.5)	51.0 (16.8)	42.5 (15.8)	**<0.001**
APS duration (months), median (IQR)	10.0 (2.0, 36.0)	10.0 (3.0, 36.0)	9.5 (2.0, 36.0)	0.292
Secondary APS, n (%)	176 (48.8)	56 (60.2)	120 (44.8)	**0.010**
Isolated thrombotic APS, n (%)	228 (63.2)	78 (83.9)	150 (56.0)	**<0.001**
Isolated obstetric APS, n (%)	101 (28.0)	0 (0.0)	101 (37.7)	**<0.001**
Thrombotic and obstetric APS, n (%)	32 (8.9)	15 (16.1)	17 (6.3)	**0.004**
Extracranial thrombotic events, n (%)	185 (51.2)	46 (49.5)	139 (51.9)	0.690
Extracranial arterial thrombotic events, n (%)	90 (24.9)	25 (26.9)	65 (24.3)	0.614
Extracranial venous thrombotic events, n (%)	130 (36.0)	30 (32.3)	100 (37.3)	0.382
Other CNS manifestations, n (%)	60 (16.6)	30 (32.3)	30 (11.2)	**<0.001**
Livedo reticularis, n (%)	13 (3.6)	8 (8.6)	5 (1.9)	**0.003**
aGAPSS, median (IQR)	10.0 (5.0, 13.0)	10.0 (7.0, 13.0)	9.0 (5.0, 13.0)	**0.022**
BMI, mean (SD)	24.4 ± 4.5	23.8 ± 4.0	24.6 ± 4.6	0.162
Smoking, n (%)	56 (15.5)	18 (19.4)	38 (14.2)	0.235
Hypertension, n (%)	109 (30.2)	47 (50.5)	62 (23.1)	**<0.001**
Diabetes, n (%)	31 (8.6)	23 (24.7)	8 (3.0)	**<0.001**
Hyperlipidemia, n (%)	55 (15.2)	23 (24.7)	32 (11.9)	**0.003**
Chronic kidney disease, n (%)	25 (6.9)	9 (9.7)	16 (6.0)	0.225
Hyperuricemia, n (%)	35 (9.7)	12 (12.9)	23 (8.6)	0.225
Atrial fibrillation, n (%)	0 (0)	0 (0)	0 (0)	1.000
aβ2GPIs, n (%)	247 (68.4)	62 (66.7)	185 (69.0)	0.673
aCL, n (%)	253 (70.1)	69 (74.2)	184 (68.7)	0.315
LA, n (%)	235 (65.1)	64 (68.8)	171 (63.8)	0.659
Triple aPL positivity, n (%)	125 (34.6)	33 (35.5)	92 (34.3)	0.840
Platelet (×10^9^/L), median (IQR)	151.4 (80.8, 215.5)	121.0 (58.0, 202.9)	165.5 (95.3, 217.8)	**0.027**
Thrombocytopenia, n (%)	143 (39.6)	46 (49.5)	97 (36.2)	**0.024**
PT (s), median (IQR)	11.5 (10.4, 13.3)	11.7 (10.6, 13.5)	11.4 (10.4, 13.3)	0.433
APTT (s), median (IQR)	34.0 (29.5, 48.9)	35.6 (29.6,51.4)	33.4 (29.4,47.4)	0.322
D-Dimer (ng/mL), median (IQR)	298.0 (110.9, 703.5)	218.0 (97.5, 553.5)	325.5 (123.0, 746.0)	0.050
Low C3, n (%)	171 (47.4)	52 (55.9)	119 (44.4)	0.055
Low C4, n (%)	341 (94.5)	90 (96.8)	251 (93.7)	0.257
Antiplatelet drugs, n (%)	136 (37.7)	38 (40.9)	98 (36.6)	0.462
Anticoagulants, n (%)	203 (56.2)	45 (48.4)	158 (59.0)	0.077
Both antiplatelet drugs and anticoagulants, n (%)	69 (19.1)	13 (14.0)	56 (20.9)	0.144
HCQ, n (%)	241 (66.8)	49 (52.7)	192 (71.6)	**0.001**
Statins, n (%)	34 (9.4)	16 (17.2)	18 (6.7)	**0.003**
Immunosuppressants, n (%)	187 (51.8)	55 (59.1)	132 (49.3)	0.100

Categorical variables are described as n (%), and continuous variables as their mean (standard deviation) or median (interquartile range), where n = number of patients, the *p* values less than 0.05 were showed in bold. APS: antiphospholipid syndrome, CNS: central nervous system, aGAPSS: the adjusted global antiphospholipid syndrome score, BMI: body mass index, aβ2GPIs: anti-β2 glycoprotein I antibodies, aCL: anticardiolipin antibody, LA: lupus anticoagulant, aPL: antiphospholipid antibody, PT: prothrombin time, APTT: activated partial thromboplastin time, C3: Complement 3, C4: Complement 4, HCQ: hydroxychloroquine.

**Table 2:** Univariate logistic regression analyses of factors associated with stroke.

Variables	OR	95%CI	*p*
Male gender	1.838	(1.080, 3.129)	**0.025**
Age (years)	1.031	(1.017, 1.047)	**˂** **0.001**
APS duration (months)	1.002	(0.998, 1.005)	0.402
Secondary APS	1.867	(1.155, 3.017)	**0.011**
Extracranial thrombotic events	0.908	(0.567, 1.456)	0.690
Extracranial arterial thrombotic events	1.148	(0.671, 1.964)	0.614
Extracranial venous thrombotic events	0.800	(0.485, 1.319)	0.382
Other CNS involvements	3.778	(2.121, 6.729)	**˂** **0.001**
aGAPSS	1.068	(1.006, 1.135)	**0.032**
BMI	0.961	(0.909, 1.016)	0.162
Smoking	1.453	(0.783, 2.696)	0.237
Hypertension	3.395	(2.068, 5.574)	**˂** **0.001**
Diabetes	10.679	(4.579, 24.901)	**˂** **0.001**
Hyperlipidemia	2.423	(1.332, 4.409)	**0.004**
Chronic kidney disease	1.687	(0.719, 3.961)	0.229
Hyperuricemia	1.578	(0.752, 3.314)	0.228
Livedo reticularis	4.951	(1.577, 15.538)	**0.006**
aβ2GPIs	0.897	(0.543, 1.484)	0.673
aCL	1.312	(0.771, 2.233)	0.316
LA	1.252	(0.756, 2.073)	0.383
Triple aPL positivity	1.052	(0.642, 1.724)	0.840
Thrombocytopenia	1.725	(1.071, 2.779)	**0.025**
Low C3	1.588	(0.988, 2.554)	0.056
Low C4	2.032	(0.582, 7.097)	0.267
Antiplatelet drugs	1.199	(0.740, 1.942)	0.462
Anticoagulants	0.653	(0.406, 1.048)	0.078
Both antiplatelet drugs and anticoagulants	0.615	(0.319, 1.185)	0.147
HCQ	0.441	(0.271, 0.717)	**0.001**
Statins	2.886	(1.404, 5.931)	**0.004**
Immunosuppressants	1.491	(0.925, 2.405)	0.101

The *p* values less than 0.05 were showed in bold. APS: antiphospholipid syndrome, CNS: central nervous system, aGAPSS: the adjusted global antiphospholipid syndrome score, BMI: body mass index, aβ2GPIs: anti-β2 glycoprotein I antibodies, aCL: anticardiolipin antibody, LA: lupus anticoagulant, aPL: antiphospholipid antibody, C3: Complement 3, C4: Complement 4, HCQ: hydroxychloroquine.

**Table 3:** Multivariate logistic regression analyses of factors associated with stroke.

	Model 1			Model 2		
	OR	95%CI	*p*	OR	95%CI	*p*
Age (years)	1.005	(0.987, 1.024)	0.566	1.015	(0.999, 1.032)	0.073
Male gender	1.634	(0.877, 3.043)	0.107	1.716	(0.929, 3.169)	0.085
Secondary APS	1.463	(0.828, 2.588)	0.190	1.381	(0.788, 2.421)	0.260
Other CNS involvements	1.929	(0.971, 3.835)	0.061	1.973	(1.000, 3.894)	0.050
aGAPSS	/	/	/	1.028	(0.959, 1.102)	0.437
Hypertension	2.201	(1.184, 4.089)	**0.013**	/	/	/
Hyperlipidemia	1.305	(0.632, 2.694)	0.471	/	/	/
Diabetes	6.185	(2.441, 15.676)	**<0.001**	6.004	(2.385, 15.111)	<0.001
Livedo reticularis	5.027	(1.341, 18.851)	**0.017**	5.338	(1.491, 19.106)	**0.010**
Thrombocytopenia	1.494	(0.864, 2.583)	0.151	1.526	(0.888, 2.625)	0.126
HCQ	0.589	(0.337, 1.029)	0.063	0.549	(0.316, 0.952)	**0.033**
Statins	2.027	(0.884, 4.646)	0.095	2.135	(0.940, 4.874)	0.070

The *p* values less than 0.05 were showed in bold. APS: antiphospholipid syndrome, CNS: central nervous system, aGAPSS: the adjusted global antiphospholipid syndrome score, HCQ: hydroxychloroquine.

**Table 4:** Univariate logistic regression analyses of predictors of stroke recurrence in APS.

	Univariate Analysis		
	HR	95% CI	*p*
Age at diagnosis (years)	0.966	(0.927, 1.007)	0.101
Male gender	1.303	(0.350, 4.855)	0.693
Disease duration (months)	1.005	(0.997, 1.013)	0.257
Secondary APS	0.507	(0.143, 1.800)	0.293
Thrombotic APS	0.457	(0.106, 1.971)	0.294
Extracranial thrombotic events	1.260	(0.356, 4.457)	0.720
Extracranial arterial thrombotic events	0.570	(0.114, 2.841)	0.493
Extracranial venous thrombotic events	1.900	(0.530, 6.810)	0.324
Other CNS involvements	0.764	(0.188, 3.112)	0.707
aGAPSS	1.038	(0.887, 1.214)	0.645
Obesity (BMI ≥ 30)	1.540	(0.163, 14.550)	0.706
Smoking	1.675	(0.397, 7.071)	0.483
Hypertension	0.794	(0.224, 2.807)	0.720
Diabetes	0.646	(0.129, 3.231)	0.594
Hyperlipidemia	1.895	(0.500, 7.173)	0.347
Chronic kidney disease *	0	/	0.999
Hyperuricemia	1.600	(0.302, 8.490)	0.581
Livedo reticularis	2.815	(0.493, 16.087)	0.245
Thrombocytopenia	0.082	(0.010, 0.672)	**0.020**
aβ2GPIs	1.383	(0.340, 5.625)	0.651
aCL	0.565	(0.150, 2.130)	0.399
LA	2.209	(0.446, 10.941)	0.332
Triple aPL positivity	1.607	(0.451, 5.732)	0.465
Low C3	0.405	(0.110, 1.492)	0.174
Low C4	0.250	(0.021, 3.012)	0.275
Antiplatelet drugs	1.237	(0.349, 4.390)	0.742
Anticoagulants	2.026	(0.551, 7.457)	0.288
Both antiplatelet drugs and anticoagulants	2.700	(0.613, 11.892)	0.189
HCQ	1.667	(0.453, 6.131)	0.442
Immunosuppressants	0.808	(0.228, 2.867)	0.742
Statins	1.079	(0.210, 5.545)	0.927

* All of the 11 recurrent stroke cases were without CKD. The *p* values less than 0.05 were showed in bold. APS: antiphospholipid syndrome, CNS: central nervous system, aGAPSS: the adjusted global antiphospholipid syndrome score, BMI: body mass index, aβ2GPIs: anti-β2 glycoprotein I antibody, aCL: anticardiolipin antibody, LA: lupus anticoagulant, aPL: antiphospholipid antibody, C3: Complement 3, C4: Complement 4, HCQ: hydroxychloroquine.

## Data Availability

The datasets used and/or analyzed during the present study are available from the corresponding authors upon reasonable request.
